# Alcohol and Substance Use After Bariatric Surgery: Nutritional Risks and Clinical Implications in Long-Term Postoperative Care

**DOI:** 10.3390/nu18060932

**Published:** 2026-03-16

**Authors:** Martín Campuzano-Donoso, Claudia Reytor-González, Gerardo Sarno, Martha Montalvan, Luigi Barrea, Giovanna Muscogiuri, Ludovica Verde, Giuseppe Annunziata, Daniel Simancas-Racines

**Affiliations:** 1 Facultad de Ciencias de la Salud y Bienestar Humano, Universidad Tecnológica Indoamérica, Ambato 180150, Ecuadorclaudiareytor@gmail.com (C.R.-G.); 2 San Giovanni di Dio e Ruggi D’Aragona” University Hospital, Scuola Medica Salernitana, 84131 Salerno, Italy; 3 Escuela de Medicina, Universidad Espíritu Santo, Samborondón 0901952, Ecuador; 4 Department of Psychology and Health Sciences, Università Telematica Pegaso, Centro Direzionale F2, 80143 Naples, Italy; 5 Centro Italiano per la Cura e il Benessere del Paziente con Obesità (C.I.B.O.), Unità di Endocrinologia, Diabetologia e Andrologia, Dipartimento di Medicina Clinica e Chirugia, Università degli Studi di Napoli Federico II, 80131 Naples, Italy; 6 Division of Endocrinology, Department of Medicine, The University of Arizona College of Medicine, Tucson, AZ 85724, USA; 7 Dipartimento di Medicina Clinica e Chirurgia, Unità di Endocrinologia, Diabetologia ed Andrologia, Università degli Studi di Napoli Federico II, 80131 Naples, Italy; 8 Cattedra Unesco “Educazione Alla Salute E Allo Sviluppo Sostenibile”, Università degli Studi di Napoli Federico II, 80131 Naples, Italy; 9 Department for the Promotion of Human Sciences and Quality of Life, San Raffaele Open University, 00166 Rome, Italy; giuseppe.annunziata@uniroma5.it

**Keywords:** bariatric surgery, alcohol-related disorders, substance-related disorders, risk factors, nutritional deficiencies, postoperative care

## Abstract

Metabolic and bariatric surgery (MBS) has evolved into a highly effective neurohormonal intervention for severe obesity; however, it introduces unique long-term vulnerabilities, particularly regarding alcohol (AUD) and substance use disorders (SUD). This review synthesizes the epidemiological, pharmacokinetic, and neurobiological drivers of postoperative substance misuse. Procedures like Roux-en-Y gastric bypass (RYGB) radically alter ethanol metabolism, eliminating first-pass metabolism and accelerating gastric emptying, while simultaneously recalibrating reward pathways, creating a “reward gap” that facilitates addiction transfer. These physiological shifts exacerbate critical micronutrient deficiencies (thiamine, B12, iron), increase the risk of post-bariatric hypoglycemia, and correlate with higher rates of liver cirrhosis and suicide. Furthermore, substance use is a primary driver of suboptimal weight loss trajectories and weight regain. Mitigation requires a lifelong, multidisciplinary framework involving preoperative risk stratification, validated screening (e.g., AUDIT-C), and targeted nutritional supplementation to safeguard the long-term metabolic and psychological benefits of MBS.

## 1. Introduction

Over the past decades, metabolic and bariatric surgery (MBS) has undergone a major conceptual shift, evolving from a primarily restrictive, mechanical intervention to a therapy recognized for its complex neurohormonal and metabolic effects [1,2]. In the context of obesity as a global public health crisis and a leading cause of preventable morbidity and mortality, MBS has emerged as the most evidence-supported and consistently effective treatment for achieving sustained weight loss and improving obesity-related comorbidities such as Type 2 Diabetes (T2D), cancer, metabolic dysfunction-associated steatotic liver disease (MASLD), polycystic ovary syndrome (PCOS), hypertension, sarcopenia, and obstructive sleep apnea [3,4,5,6,7]. Since the National Institutes of Health (NIH) issued its 1991 consensus statement on gastrointestinal surgery for severe obesity, substantial advances in clinical experience and research have transformed the understanding of both obesity and MBS [8]. Reflecting this evolution, contemporary guidelines, including the 2022 joint statement from the American Society for Metabolic and Bariatric Surgery (ASMBS) and the International Federation for the Surgery of Obesity and Metabolic Disorders (IFSO), have broadened surgical indications, recommending MBS for individuals with a body mass index (BMI) ≥35 kg/m^2^ regardless of comorbidities, and for those with BMI 30–34.9 kg/m^2^ who fail to achieve sustained improvement with non-surgical therapies [8,9], and recognizing metabolic bariatric surgery as an appropriate treatment option for selected adolescents with severe obesity [10].

Globally, obesity prevalence has risen at an unprecedented rate, with more than 650 million adults now classified as individuals with obesity and projections indicating continued growth across both developed and developing regions [11,12,13,14]. On the other hand, the utilization of bariatric surgery has increased substantially, with hundreds of thousands of procedures performed annually worldwide, reflecting its growing acceptance as a frontline therapy for severe obesity and metabolic disease [15,16]. As surgical volumes expand the long-term metabolic, behavioral, and nutritional consequences of MBS have become increasingly central to chronic patient management [17,18].

Despite their benefits, procedures such as Roux-en-Y gastric bypass (RGYB) and sleeve gastrectomy (SG) entail significant anatomical and physiological changes that carry long-term clinical implications [19]. Among the most significant is the emergence of alcohol use disorder (AUD) and other substance use disorders (SUD) in the postoperative period [20]. While weight loss generally improves health-related quality of life, a subset of patients experiences a late-onset escalation in hazardous substance consumption, often manifesting several years after the initial procedure [21]. A systematic review found that bariatric surgery is associated with a delayed increase in substance use disorders, particularly alcohol, typically emerging about two years postoperatively, often in patients without prior substance use, with higher risk observed among younger males, individuals with psychiatric histories, and those undergoing bypass procedures, underscoring the need for careful patient selection and long-term follow-up [21].

The pathophysiology of post-bariatric substance misuse is multifactorial, involving radical shifts in ethanol pharmacokinetics, alterations in mesolimbic reward pathways, and the potential for addiction transfer as food-based coping mechanisms are rendered physically restricted, although this concept remains debated and not definitively established [22,23,24]. These behavioral changes arise in a physiological context already affected by postoperative malabsorption, thereby amplifying the risk of serious nutritional deficiencies, particularly in thiamine (B1), vitamin B12, and iron [25]. Furthermore, the interaction between chronic alcohol use and the post-bariatric state exacerbates clinical complications such as post-bariatric hypoglycemia (PBH), marginal ulcers, and alcohol-related liver disease (ALD) [26,27,28,29,30].

This review aims to synthesize current evidence on the epidemiology, mechanisms, and risk factors for alcohol and substance use following bariatric surgery, with a particular focus on the associated nutritional consequences. We further seek to examine the clinical implications for long-term postoperative care and to highlight screening, monitoring, and multidisciplinary management strategies to mitigate risk and optimize patient outcomes.

## 2. Methodology

This narrative review was conducted through a structured literature search of PubMed, Scopus, and other major biomedical databases from inception through January 2025. Search terms were combined using Boolean operators and included “bariatric surgery,” “metabolic bariatric surgery,” “alcohol use disorder,” “substance use,” “addiction transfer,” “postoperative alcohol metabolism,” “weight regain,” “nutritional deficiencies,” and “post-bariatric complications.” Priority was given to clinical studies evaluating postoperative bariatric populations, including cohort studies, randomized trials, and systematic reviews. When direct evidence in post-bariatric cohorts was limited, high-quality studies in general obesity or addiction populations were included to provide mechanistic and clinical context. Reference lists of relevant articles and reviews were manually screened to identify additional sources, and selected grey literature (including conference proceedings and professional society statements) was reviewed when appropriate. Given the heterogeneity of study designs and the limited availability of randomized trials addressing alcohol and substance use outcomes after metabolic bariatric surgery, a narrative synthesis approach was adopted rather than a formal systematic review.

## 3. Alcohol and Substance Use in the Post-Bariatric Population

### 3.1. Epidemiological Surveillance and Long-Term Trends

The association between MBS and increased alcohol-related complications is supported by robust longitudinal evidence. A long-term prospective cohort with follow-up up to 35 years of Swedish patients with obesity demonstrates a strong association between MBS and alcohol-related harm. Compared with matched controls, patients undergoing gastric bypass exhibited more than a five-fold higher risk of AUD (HR_adj 5.07; 95% CI 3.11–8.25), while elevated risks were also observed after vertical banded gastroplasty and gastric banding. Importantly, alcohol-related mortality was significantly increased following gastric bypass (sub-HR_adj 6.18; 95% CI 2.48–15.40) and vertical banded gastroplasty, highlighting that MBS, particularly bypass procedures, is associated with both incident AUD and excess alcohol-related death [31]. Consistent with these long-term findings, prospective and temporal analyses indicate that the risk of alcohol use disorder after bariatric surgery follows a delayed trajectory. In a large cohort study of 1945 patients, King et al. observed no increase in AUD during the first postoperative year, but reported a significant rise by the second year (7.3% vs. 9.6%, *p* = 0.01), suggesting a period of relative early stability. This initial phase may reflect strict adherence to dietary recommendations and the early “honeymoon period” of rapid weight loss [32,33,34]. However, longer-term data further underscore this progressive risk, with Bramming et al. demonstrating that the greatest vulnerability emerges beyond five years post-surgery, when bariatric patients exhibit a six- to seven-fold higher risk of AUD compared with non-surgical controls [35]. Some authors agree that this risk continues to escalate, indicating that alcohol-related issues continue to rise over time, with approximately 20% of RYGB patients reporting significant symptoms of alcohol problems by five to seven years after surgery [20]. These findings highlight that the risk of alcohol use disorder after bariatric surgery is both delayed and progressive, underscoring the importance of long-term surveillance and targeted preventive strategies for at-risk patients.

When considered collectively, large longitudinal cohorts reveal a consistent procedure-specific gradient in alcohol-related risk [31,36]. RYGB is associated with the highest relative risk of incident alcohol use disorder, with adjusted hazard ratios typically ranging between 4 and 5 in long-term follow-up studies, whereas vertical banded gastroplasty demonstrates intermediate elevations and purely restrictive procedures such as gastric banding show minimal or no significant increase compared with non-surgical controls [36]. Importantly, the emergence of postoperative alcohol misuse follows a delayed trajectory, with incidence peaking two to five years after surgery and continuing to rise beyond five years in some cohorts [32,35,37]. This procedural and temporal pattern suggests that surgery-specific physiological changes may contribute to postoperative vulnerability, although psychosocial and behavioral factors also likely play important roles [23].

### 3.2. Pharmacokinetic Mechanisms of Enhanced Sensitivity

The primary physiological driver for increased alcohol vulnerability is the radical alteration of ethanol metabolism characterized by accelerated absorption, reduced first-pass metabolism, and higher peak blood alcohol concentrations after procedures such as RGYB and sleeve gastrectomy (SG) [37]. In a non-surgical individual, alcohol undergoes significant first-pass metabolism (FPM) in the stomach, where gastric alcohol dehydrogenase (ADH) begins the oxidative breakdown of ethanol [38]. Bariatric procedures disrupt this protective process by reducing gastric volume, shortening ethanol contact time with gastric alcohol dehydrogenase, and rapidly delivering ethanol to the proximal small intestine, where absorption is more efficient [38,39,40].

A central component of this altered pharmacokinetic profile is the disruption of FPM: by bypassing much of the stomach and duodenum in RYGB or markedly reducing gastric contact time in SG, ethanol is rapidly delivered to the small intestine, effectively removing the protective role of gastric ADH [39,40]. This near-elimination of FPM, previously observed after gastrectomy and gastric bypass, results in higher blood alcohol concentrations, increased systemic bioavailability, and greater susceptibility to alcohol-related harm [41]. Supporting this, a cross-sectional study of women who had undergone SG within the previous five years demonstrated a 34% increase in alcohol bioavailability compared with non-surgical controls, indicating that the reduction or loss of gastric FPM plays a significant role in increasing systemic ethanol bioavailability in women [42].

When pharmacokinetic profiles are compared across bariatric procedures, a consistent pattern emerges. Controlled studies using arterialized blood sampling have demonstrated that, after RYGB, alcohol reaches the systemic circulation more rapidly and produces approximately two-fold higher peak blood alcohol concentrations, accompanied by greater subjective intoxication, compared with non-surgical controls [43]. Importantly, sleeve gastrectomy produces a comparable acceleration in ethanol absorption and elevation in peak blood alcohol concentrations when assessed using direct blood measurements. A prospective alcohol challenge study comparing SG, RYGB, and presurgical controls demonstrated that both SG and RYGB resulted in approximately two-fold higher peak blood alcohol concentrations and greater subjective intoxication relative to controls, while breathalyzer-derived estimates underestimated peak systemic exposure by nearly 30%, frequently missing the true C_max after surgery [44]. In a longitudinal observational cohort of sleeve gastrectomy patients, the prevalence of high-risk alcohol use increased markedly in the first postoperative year, with nearly one in five participants developing new high-risk drinking behavior, suggesting that the pharmacokinetic alterations observed after SG may translate into clinically meaningful increases in postoperative alcohol misuse [45]. A controlled alcohol challenge study further demonstrated that although RYGB and SG produced markedly accelerated absorption and peak BACs exceeding legal driving limits, nearly one-third of women reported minimal sedative effects, suggesting that reduced subjective alcohol sensitivity, despite high systemic exposure, may represent an independent vulnerability factor for postoperative AUD [46]. In contrast, purely restrictive procedures such as adjustable gastric banding, which preserve gastric mucosal surface area and first-pass metabolism, do not demonstrate significant changes in systemic alcohol exposure [47,48]. Consistent with this, in the prospective Swedish Obese Subjects (SOS) study with up to 22 years of follow-up, gastric banding was not associated with an increased risk of alcohol abuse diagnoses or alcohol-related problems compared with matched controls, whereas gastric bypass showed a nearly five-fold higher hazard of alcohol abuse [36]. These findings indicate that reduction in functional gastric reservoir and mucosal contact area, rather than intestinal bypass alone, is the critical determinant of enhanced ethanol bioavailability after metabolic surgery [44,49]. Importantly, available pharmacokinetic studies are limited by small sample sizes and predominantly female cohorts, which may restrict generalizability.

These changes are closely linked to accelerated gastric emptying and reduced gastric reservoir capacity following surgery. The loss of pyloric regulation and marked reduction in gastric volume allow liquids to pass rapidly into the small intestine, producing a “dumping” effect in which ethanol is delivered directly to the jejunum for rapid systemic absorption [40,50]. Faster gastric emptying and diminished gastric reservoir function further shorten ethanol contact time with gastric ADH and increase the rate and extent of absorption. Pharmacokinetic studies demonstrate that many orally administered substances exhibit shorter time to peak concentration (T_max) and higher peak levels (C_max) after bariatric surgery, consistent with accelerated intestinal delivery [51,52]. This phenomenon has been confirmed using paracetamol as a validated marker of gastric emptying, with comparable effects observed after both RYGB and SG, indicating that both procedures similarly enhance absorption kinetics [53].

Together, these interconnected changes result in markedly enhanced systemic ethanol bioavailability after bariatric surgery. Rapid gastric transit and near-elimination of FPM result in ethanol entering the systemic circulation in a manner resembling intravenous delivery, producing disproportionately high peak blood alcohol concentrations from relatively small amounts [40]. In addition, anatomical and metabolic changes following malabsorptive procedures modify drug handling, and intestinal enzyme expression, further influencing oral bioavailability of various orally administered agents [54]. Most pharmacokinetic studies demonstrate faster absorption after surgery due to reduced gastric volume and accelerated emptying, leading to earlier and greater systemic exposure [55]. Although postoperative reductions in liver size have been proposed as a potential contributor to altered ethanol handling, recent evidence suggests that alcohol elimination rates are associated with fat-free mass, a proxy for metabolic tissue volume, and that bariatric surgery per se does not independently impair hepatic ethanol elimination when body composition is accounted for [56].

### 3.3. Neurobiological Vulnerability and Reward Signaling

Beyond the mechanical changes in absorption, MBS induces profound alterations in the neurobiological pathways governing reward and satiety [57]. The mesolimbic dopaminergic system, which regulates the reinforcing properties of both food and drugs, is modulated by gut hormones that shift dramatically after surgery [57,58,59]. Functional neuroimaging studies in postoperative patients have shown altered activation of the nucleus accumbens and ventral tegmental area (VTA) in response to food cues, suggesting a recalibration of reward sensitivity that may predispose some individuals to seek alternative sources of hedonic stimulation, including alcohol [60,61]. However, direct longitudinal human studies linking these neural changes to subsequent development of alcohol use disorder remain limited, and much of the mechanistic framework is extrapolated from animal models and cross-sectional imaging data [62,63,64,65,66]. Additionally, genetic and epigenetic factors may modulate individual susceptibility, as polymorphisms in dopamine receptor genes (e.g., *DRD2*) and variations in reward-related neural circuits have been associated with both obesity and substance use behaviors [67,68].

Ghrelin, the “hunger hormone” primarily produced in the gastric fundus, is often elevated in fasting plasma of patients with obesity and exhibits a blunted postprandial decline compared with lean individuals [69]. Following bariatric procedures such as RYGB and SG, circulating ghrelin levels typically decrease markedly, reflecting both anatomical resection and altered regulatory signaling [70,71]. While ghrelin levels generally decrease shortly after RYGB, longer-term studies report variable findings, with fasting levels often returning toward baseline over time [72,73]. Interestingly, some patients regain the previously absent postprandial decline, suggesting partial restoration of normal ghrelin regulation, as supported by a meta-analysis showing short-term decreases but long-term increases in fasting ghrelin after RYGB [74]. However, residual ghrelin signaling may exert a disproportionately strong effect on reward pathways postoperatively [75]. Animal models demonstrate that ghrelin can reinstate alcohol-seeking behaviors and that changes in growth hormone secretagogue receptor (GHSR) activity influence tonic dopamine firing in the VTA, thereby promoting ethanol intake [76]. These findings suggest that even reduced ghrelin levels may have amplified effects on reward sensitivity in the context of surgically altered neurophysiology. On the other hand, while these findings provide a plausible biological link between altered gut signaling and alcohol-seeking behavior, translational human data specifically demonstrating that postoperative ghrelin dynamics predict incident alcohol misuse are currently sparse.

GLP-1 and PYY, anorexigenic hormones that increase sharply after gastric bypass, contribute further to the complex modulation of reward [77]. GLP-1 promotes satiety primarily through the hypothalamic arcuate nucleus by activating anorexigenic pro-opiomelanocortin neurons and inhibiting orexigenic neuropeptide Y/Agouti-related peptide (AgRP) neurons via GLP-1 receptor signaling [78,79]. In contrast, PYY is a hormone produced by the gut that suppresses appetite [80]. While GLP-1 and peptide YY (PYY) suppress food intake by promoting satiety, their exaggerated postoperative surge has been hypothesized to create a relative shift in reward valuation for caloric intake, potentially increasing sensitivity to alternative hedonic stimuli such as alcohol to compensate [81,82,83]. Interestingly, variability exists among patients; elevated GLP-1 levels have been shown in some studies to induce mild alcohol aversion, highlighting interindividual differences in hormonal and behavioral responses [81].

**Figure 1:**
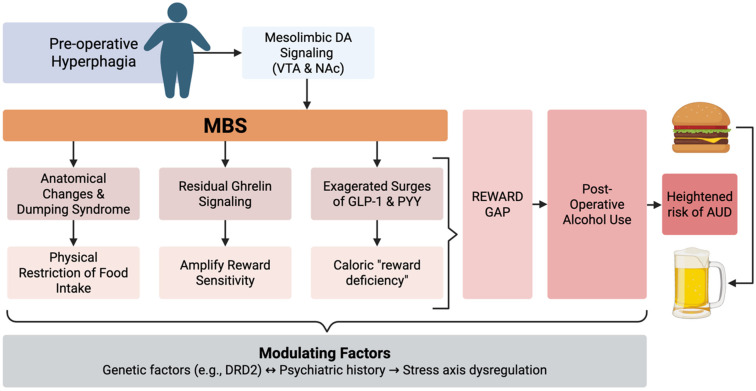
Pathophysiological mechanism of addiction transfer following (MBS). This flowchart illustrates the transition from pre-operative hyperphagia to post-operative alcohol use. Pre-operatively, food activates mesolimbic dopamine (DA) signaling in the ventral tegmental area (VTA) and nucleus accumbens (NAc) as a coping mechanism. Following MBS, anatomical changes and dumping syndrome physically restrict food intake. Simultaneously, profound hormonal shifts occurs: residual ghrelin signaling may amplify reward sensitivity, while exaggerated surges of GLP-1 and PYY contribute to a caloric “reward deficiency” state. This creates a “Reward Gap” where the recalibrated brain seeks hedonic stimulation, but food is no longer a viable option. Consequently, patients may substitute alcohol, which is rapidly absorbed and rewarding, leading to addiction transfer and a heightened risk of Alcohol Use Disorder (AUD). This process is further modulated by genetic factors (e.g., *DRD2*), psychiatric history, and stress-axis dysregulation. Although this conceptual model depicts a transition from altered food reward to increased vulnerability to alcohol use, the emergence of postoperative alcohol misuse typically occurs with a delay of 1–2 years rather than immediately after surgery. This temporal pattern likely reflects the progressive nature of neurohormonal adaptation, psychosocial adjustment, and relaxation of early postoperative dietary restrictions. Early postoperative phases are characterized by intensive medical monitoring and dietary limitations, whereas later phases involve stabilization of weight loss, evolving reward signaling, and greater behavioral autonomy, which together may unmask susceptibility to substance use. Accordingly, the proposed “reward gap” should be interpreted as a dynamic and evolving vulnerability rather than an immediate substitution phenomenon [57,60,70,76,78,79,84,88,89,90,91].

Reflecting the concept of addiction transfer, defined as the substitution of compulsive eating with other addictive behaviors after surgery, bariatric procedures may redirect maladaptive coping from hyperphagia to alcohol or substance use, as eating is physically restricted without addressing underlying vulnerability to addiction (Figure 1) [84]. Notably, not all patients demonstrate substitution behaviors, and the construct of “addiction transfer” remains debated, with some studies suggesting that preexisting vulnerability traits rather than surgical factors alone may drive postoperative substance misuse [85,86]. Patients who previously used binge eating to manage negative emotions often experience physical limitations that prevent overeating due to gastric restriction or dumping syndrome [24]. Alcohol, being calorie-dense and rapidly rewarding, becomes a readily accessible substitute, which may explain the observed increase in postoperative alcohol use [87]. Clinical data indicate that patients with preexisting psychiatric comorbidities, histories of substance use, or maladaptive coping strategies are particularly susceptible to this phenomenon [88,89].

Beyond gut hormones and behavioral shifts, other neurobiological and psychosocial factors may contribute to heightened AUD risk. Postoperative changes in stress-axis regulation, including blunted cortisol response and altered hypothalamic–pituitary–adrenal (HPA) activity, can enhance vulnerability to addictive behaviors [24,92]. Sleep disturbances, common after rapid weight loss, may further dysregulate reward circuitry and impulse control [93,94]. Social and environmental factors, such as reduced support networks or exposure to permissive drinking contexts, interact with these biological changes to shape long-term risk profiles [89,95].

Taken together, these findings underscore that the vulnerability to alcohol use disorder after bariatric surgery is multifactorial, arising from an intricate interplay of hormonal, neurobiological, and psychosocial mechanisms. Understanding these pathways is critical for identifying at-risk individuals and designing targeted interventions, including early behavioral counseling, hormone-modulating therapies, and longitudinal monitoring of reward-seeking behaviors, to mitigate the potential for addiction transfer and alcohol-related harm.

### 3.4. Trends in Opioid, Cannabis and Nicotine Use

While ethanol remains the most prevalent substance of concern, the use of opioids, cannabis, and nicotine in the bariatric population is gaining clinical attention, though the quality of evidence varies across these domains [96,97]. It is well-documented that patients with severe obesity have a high prevalence of chronic pain (up to 254% higher rates in Class III obesity than in normal BMI groups), leading to high rates of preoperative opioid prescriptions [98]. Thus, a substantial proportion of patients enter surgery with established patterns of opioid use, which may persist despite improvements in weight-related musculoskeletal symptoms [98].

Robust longitudinal data confirm that preoperative opioid use is a primary predictor of postoperative use; consumption does not decline after surgery and may, in some patients, increase [99]. A large multicenter retrospective cohort of over 11,000 bariatric patients provides high-quality evidence for this trend: 77% of individuals who were chronic opioid users preoperatively continued opioid use one year after surgery, with a significant increase in mean daily morphine equivalents despite substantial weight loss. Notably, reductions in BMI, as well as the presence of depression or chronic pain, did not significantly modify postoperative opioid use, underscoring the persistence of opioid dependence following bariatric surgery [100]. Moreover, new-onset opioid use and misuse have been documented in previously opioid-naïve patients, remaining as largely observational and mechanistic evidence and suggesting that altered reward processing, and unresolved psychosocial stressor mechanisms may contribute to postoperative vulnerability [101,102]. Persistent opioid exposure is clinically relevant, as it is associated with impaired weight loss outcomes, higher rates of gastrointestinal dysmotility, increased fall risk, and poorer quality of life [103].

The impact of cannabis on bariatric outcomes presents a more complex and less certain risk profile. While its prevalence is reported at 6–8%, its long-term effect on weight maintenance is not yet fully established [97]. Although commonly used to manage pain, anxiety, or sleep disturbances, chronic cannabis use has been linked to increased appetite and food cravings (“the munchies”), potentially undermining dietary adherence and long-term weight maintenance, but high-quality longitudinal studies confirming this link are currently limited [104]. Perioperatively, cannabis users may require higher doses of opioid analgesics, complicating pain control strategies and potentially increasing exposure to opioids [67]. Additionally, abrupt cessation prior to surgery can precipitate withdrawal symptoms, including irritability, insomnia, nausea, and weight loss, which may impair early postoperative recovery and nutritional intake [97,105]. Some studies further suggest associations between regular cannabis use and reduced adherence to follow-up visits, micronutrient supplementation, and structured dietary plans, although high-quality longitudinal studies remain limited [106,107].

In contrast to cannabis, the link between nicotine use and adverse surgical outcomes is firmly established. Although direct data in post-bariatric cohorts are limited, smoking prevalence among surgical candidates remains substantial, and evidence from bariatric and non-bariatric gastrointestinal surgery consistently links continued nicotine exposure to adverse outcomes, including marginal ulceration, anastomotic complications, impaired wound healing, and increased risk of gastrointestinal bleeding [108,109,110,111,112]. From a behavioral perspective, alcohol and other drugs, including nicotine, may substitute for overeating after weight loss surgery, as restrictive anatomy limits prior coping mechanisms based on food intake [113]. From a nutritional perspective, nicotine suppresses appetite, alters taste perception, and has been associated with reduced intake of key micronutrients, while also contributing to bone loss and sarcopenia, conditions already prevalent after bariatric surgery [114,115,116,117]. While smoking has been associated with higher rates of weight regain, poorer quality of life, and frequent relapse following preoperative cessation, particularly among individuals with psychiatric comorbidities or concurrent substance use, the evidence base for nicotine as a chronic metabolic liability is less robust than the evidence for its immediate perioperative complications [118,119,120,121]. Collectively, these findings suggest that nicotine use should be viewed not only as a perioperative risk factor but also as a chronic metabolic and nutritional liability requiring long-term monitoring and targeted intervention.

These data indicate that non-alcohol substances, including opioids, cannabis, and nicotine, may independently and synergistically compromise metabolic outcomes, nutritional status, and postoperative safety. While the surgical risks of nicotine and the persistence of opioid use are supported by strong, large-scale data, the long-term behavioral impacts of cannabis and the metabolic consequences of nicotine represent emerging fields with limited evidence. Table 1 summarizes the prevalence, proposed mechanisms, and clinical consequences of these substances in the bariatric population, highlighting the need for routine screening and longitudinal monitoring beyond alcohol alone.

## 4. Nutritional Risks Associated with Alcohol and Substance Use

### 4.1. Exacerbation of Micronutrient Deficiencies

MBS patients are inherently at risk for micronutrient deficiencies due to reduced gastric acid, bypass of the duodenum and proximal jejunum (in RYGB), and reduced dietary intake [123,124]. Alcohol acts as a powerful disruptor of nutritional status by impairing the intestinal absorption and subsequent utilization and storage of a wide range of essential nutrients, including macronutrients, water, vitamins, and minerals [125].

#### 4.1.1. Thiamine (Vitamin B1)

Thiamine is an essential cofactor in carbohydrate metabolism, as its active form, thiamine pyrophosphate (TPP), is required for key enzymatic steps within the Krebs cycle, including pyruvate and α-ketoglutarate dehydrogenase reactions [126]. Because thiamine is water-soluble and minimally stored in the body, its reserves can be rapidly exhausted during periods of inadequate intake or heightened metabolic demand [127]. Thiamine deficiency is a frequent early complication after bariatric surgery, particularly following RYGB and SG, as patients possess only limited body reserves, typically sufficient for just two to three weeks, which are further and rapidly depleted by alcohol consumption [128,129,130]. Ethanol inhibits the intestinal thiamine transporters (THTR-1 and THTR-2), impairs the conversion of B1 to its active diphosphate form in the liver, and increases urinary excretion [131]. Chronic alcohol use has long been recognized as a major cause of thiamine deficiency, predisposing individuals to Wernicke–Korsakoff syndrome [131]. In the post-bariatric setting, this condition may be triggered by even a single episode of heavy alcohol consumption or by a brief period of inadequate nutritional intake in combination with alcohol use [132].

Clinical consequences range from subclinical deficiency to the classic neuropsychiatric manifestations of Wernicke’s encephalopathy and Korsakoff psychosis (Table 2). Importantly, delayed recognition is common, as early symptoms may be nonspecific and masked by postoperative gastrointestinal complaints. The vulnerability of this population is illustrated by a case report of a patient who developed probable Korsakoff syndrome a decade after RYGB in the setting of alcohol use disorder and discontinuation of vitamin supplementation. The patient presented with severe memory impairment, confabulation, nystagmus, and ataxia, with MRI demonstrating mammillary body atrophy; despite parenteral and intramuscular thiamine therapy, only limited neurological recovery was observed, underscoring the potentially irreversible nature of late-stage disease when diagnosis is delayed [133].

#### 4.1.2. Cobalamin (B12) and Folate (B9)

Vitamin B12 absorption depends on gastric acid to release it from dietary proteins and on intrinsic factor (IF) for uptake in the terminal ileum. Gastric bypass drastically reduces both [138]. Alcohol induces atrophic gastritis and further compromises the secretion of IF, leading to profound B12 deficiency [139,140,141]. Folate deficiency is also common in AUD due to impaired enterohepatic circulation and reduced intestinal absorption [142]. In a cross-sectional study of 211 patients admitted for alcohol detoxification, folate deficiency was common, with 23% showing low serum folate and 7% low erythrocyte folate, underscoring that folate depletion remains a prevalent nutritional complication in patients with alcohol use disorder [143].

#### 4.1.3. Iron and Anemia

Iron deficiency is the most prevalent micronutrient complication after RYGB and SG, affecting approximately 30–60% of patients in the long term [144]. This risk is driven by bypass of the duodenum and proximal jejunum, the primary sites of iron absorption, together with reduced gastric acid, which impairs conversion of ferric to bioavailable ferrous iron, and postoperative dietary limitations that restrict intake of iron-rich foods such as red meat [25,130]. Alcohol consumption increases the risk of iron-deficiency anemia through gastrointestinal blood loss (gastritis, ulcers) and by interfering with the duodenal uptake of non-heme iron [145]. In a U.S. population study, moderate alcohol consumption (≤2 drinks/day) was associated with a lower risk of iron deficiency and iron deficiency anemia, while heavy drinking (>2 drinks/day) significantly increased the risk of iron overload [145].

Although numerous studies have independently examined micronutrient deficiencies in post-bariatric patients and the impact of alcohol or substance use on nutritional status, there remains a substantial gap in research integrating both domains. Specifically, few investigations have clinically assessed how AUD or other substance misuse interacts with post-bariatric malabsorption to exacerbate deficiencies in key micronutrients such as thiamine, vitamin B12, folate, and iron. Addressing this gap is critical, as understanding the combined effect of surgery-related malabsorption and substance use could inform targeted screening, supplementation strategies, and long-term monitoring protocols in this high-risk population.

### 4.2. Post-Bariatric Hypoglycemia (PBH) and Ethanol

Post-bariatric hypoglycemia (PBH) is a late complication, usually emerging more than one year after surgery, marked by symptomatic drops in blood glucose 1–3 h following a carbohydrate-rich meal. In some patients, these postprandial hypoglycemic episodes can produce neuroglycopenic symptoms, including confusion, loss of consciousness, and, in rare cases, seizures [146]. The mechanism involves rapid transit of simple carbohydrates into the small intestine, causing a massive spike in glucose and a subsequent exaggerated insulin response (hyperinsulinemia) [147].

As discussed in Section 3.2, bariatric surgery profoundly alters ethanol pharmacokinetics, resulting in accelerated absorption and enhanced systemic exposure. These pharmacokinetic changes intersect with glycemic regulation in clinically important ways. Alcohol consumption can markedly exacerbate post-bariatric hypoglycemia through multiple mechanisms [30]. Ethanol inhibits hepatic gluconeogenesis, reducing the liver’s ability to produce glucose from precursors such as lactate and glycerol, which normally protects against hypoglycemia during periods of high insulin [148,149]. Additionally, many alcoholic beverages, such as beer, sweet wines, and cocktails, contain simple sugars that can trigger the same hyperinsulinemic response as a meal, further lowering blood glucose [150]. Thus, the combination of rapid ethanol delivery and impaired counter-regulatory glucose production may amplify the severity and duration of hypoglycemic episodes in susceptible individuals [151]. Clinically, the neuroglycopenic symptoms of PBH, including confusion, slurred speech, and ataxia, closely resemble those of alcohol intoxication, potentially delaying recognition and timely glucose administration [152].

### 4.3. Bone Mineral Density and Vitamin D

Bariatric surgery is associated with accelerated bone loss and a heightened risk of fractures, with bone resorption occurring after both SG and more prominently after RYGB. This skeletal deterioration is driven by malabsorption of calcium and vitamin D, secondary hyperparathyroidism, and hormonal changes related to reduced adipose tissue, placing MBS patients at increased risk for osteoporosis and osteomalacia [153,154]. Rapid weight loss itself has been linked to adverse skeletal outcomes and is frequently associated with patterns of binge alcohol consumption [155]. Chronic alcohol exposure further compounds bone deterioration by exerting direct toxic effects on osteoblasts and impairing hepatic 25-hydroxylation of vitamin D, thereby disrupting calcium homeostasis [156]. The micronutrient deficiencies, previously addressed in this review, further contribute to skeletal fragility: although hypomagnesemia is most common, abnormalities such as hypocalcemia, hypercalciuria, hypophosphatemia, and hypokalemia are also well documented, along with widespread defects in renal tubular handling of minerals in individuals with chronic alcohol use [157]. Furthermore, AUD is associated with an increased fall risk, creating a dangerous combination of skeletal fragility and mechanical trauma [158].

## 5. Clinical Implications for Postoperative Care

Postoperative management after MBS is increasingly shaped by the profound physiological and behavioral changes that alter patients’ responses to alcohol and other substances [87]. Although MBS reliably produces substantial weight loss and metabolic improvement, long-term outcomes depend heavily on identifying and managing AUD and substance use disorders (SUD). These conditions not only undermine weight loss durability but also contribute to severe neurological, hepatic, and psychological complications.

### 5.1. Effects on Weight Loss Trajectories and Regain

The impact of alcohol and substance use on weight loss (WL) trajectories is one of the most immediate and visible clinical concerns in postoperative care [113]. Although MBS is the most effective evidence-based treatment for obesity, providing durable total body WL of 20% to 30%, a significant minority of patients experience suboptimal results [8]. Suboptimal WL is typically defined as achieving less than expected postoperative weight reduction, often corresponding to <20% total weight loss (TWL) or, in studies using older metrics, <40–60% excess weight loss (EWL) within the first 24 months [159]. Furthermore, WR remains a pervasive long-term challenge. Importantly, weight regain after MBS is multifactorial and also reflects postoperative physiological adaptations in gut–brain signaling and appetite-regulating hormones that may establish a new homeostatic equilibrium favoring increased hunger and energy intake, rather than solely behavioral factors [160]. Longitudinal data indicate that weight regain as a percentage of nadir weight increases steadily over time, reaching an average of 15% by five years post-nadir [161].

Alcohol consumption is a primary driver of WR due to its high caloric density and lack of nutritional value [162,163]. Beyond the direct caloric contribution, alcohol misuse is strongly associated with maladaptive eating behaviors that undermine surgical restriction and malabsorption [164]. Grazing, defined as the unplanned and repetitive consumption of small amounts of food, occurs in 17% to 47% of patients and is a robust predictor of WR [165]. Additionally, the persistence or recurrence of food addiction plays a critical role. While the prevalence of food addiction may drop from 32% preoperatively to 15% postoperatively, research suggests a “rebound” effect where symptoms re-emerge between 24 months and 10 years after surgery [166]. High food addiction symptom counts at five years post-surgery are predictive of lower total WL and decreased psychological functioning at the 10-year mark [166].

The physiological mechanism of weight regain in the context of substance use involves a complex interplay between reward signaling and homeostatic regulation [28]. Alcohol and high-glycemic foods activate similar dopaminergic pathways in the brain’s ventral tegmental area. After surgery, shifts in the gut microbial composition may enhance these hedonic signals through the microbiome–gut–brain axis, making patients more susceptible to “loss-of-control” eating when under the influence of alcohol [167,168]. This biological enhancement of reward makes substance-using patients particularly prone to the caloric surfeit required for significant weight recurrence [113].

### 5.2. Metabolic Outcomes and Type 2 Diabetes Remission

MBS induces remission of type 2 diabetes in 30–70% of patients, often through improved insulin sensitivity and beta-cell function [169]. However, alcohol disrupts glycemic control by impairing gluconeogenesis and worsening insulin resistance, threatening the durability of remission [170]. Notably, RYGB confers greater metabolic benefit than SG but also carries a substantially higher risk of AUD, creating a clinical paradox in long-term metabolic management [171]. Chronic alcohol consumption often mirrors the use of antidepressants and other psychiatric medications, which may further complicate metabolic stability [172,173].

### 5.3. Liver Disease and Alcohol-Associated Cirrhosis

The risk of end-stage liver disease (ESLD) and alcohol-associated liver disease (ALD) represents perhaps the most severe long-term clinical implication of post-bariatric alcohol use [26]. While MBS is known to improve MASLD, it simultaneously increases the liver’s vulnerability to ethanol-induced injury [174,175,176,177,178]. Population-based studies have revealed that bariatric surgery is followed by an increased incidence (HR 1.23) and significantly higher mortality (HR 1.93) in ESLD compared to non-operative care [179].

This increased vulnerability is partly related to the altered ethanol pharmacokinetics described in Section 3.2, which result in faster absorption and higher peak systemic exposure after surgery. Rather than repeating these mechanisms in detail, it is important to recognize that rapid hepatic exposure to higher ethanol concentrations may amplify hepatocellular injury over time. This effect of ethanol on the liver, combined with the metabolic stress of rapid weight loss, accelerates the progression from steatosis to cirrhosis [26].

### 5.4. Mental Health and Suicide

The psychological landscape of the post-bariatric patient is complex [180]. While many experience improved self-esteem and reduced depression in the “honeymoon” phase of weight loss, the long-term data reveal significant psychiatric vulnerabilities [181]. There is a robust association between bariatric surgery and an increased risk of suicidal ideation, self-harm, and accidental death [182]. The odds of suicide in the bariatric population are estimated to be 1.9 to 3.8 times higher than in control groups [183].

Importantly, these psychiatric comorbidities and SUD can fundamentally compromise nutritional care pathways. Depression and anxiety often impair executive function and self-efficacy, making it difficult for patients to adhere to complex daily supplementation schedules or maintain the motivation required for structured meal planning [184,185]. When patients develop new-onset AUD or SUD, potentially linked to physiological changes in reward pathways, their priorities often shift away from metabolic health, leading to missed follow-up appointments and the substitution of nutrient-dense foods for substances [186]. Furthermore, the use of GLP-1 receptor agonists, often used post-surgery to manage weight regain, has been associated with a significantly increased risk of psychiatric disorders, including a 195% higher risk of major depression [187].

### 5.5. Economic Burden and Healthcare Resource Utilization

The economic impact of alcohol and substance use in the post-bariatric population is substantial, affecting both individual patients and the broader healthcare system. Initially, MBS is associated with significant cost reductions, primarily through a 56% drop in medication expenditures for conditions like diabetes and hypertension [188]. However, these savings can be completely negated by the high costs of substance-related complications [188].

Excessive alcohol use is estimated to cost the U.S. economy $249 billion annually [189]. For individual bariatric patients, a diagnosis of AUD or SUD is linked to higher annual healthcare expenditures, approximately $14,918 higher for those with commercial insurance and $4823 higher for those on Medicaid [190]. This “financial toxicity” directly impacts nutritional adherence; patients facing high out-of-pocket costs for emergency department visits or psychiatric treatment may be forced to choose between medical bills and the purchase of high-quality protein sources or specialized bariatric vitamins [190,191,192]. These economic barriers, combined with the higher rates of inpatient hospitalizations, often lead to fragmented care where routine nutritional monitoring is deprioritized in favor of managing acute crises.

## 6. Screening, Monitoring, and Management Strategies

### 6.1. Preoperative Risk Assessment and Stratification

The preoperative period provides the most critical opportunity to identify patients who may be vulnerable to postoperative substance misuse [193]. Current recommendations stress the importance of a comprehensive evaluation that includes detailed assessment of current and prior substance use, as well as family history of addiction, reflecting the contribution of genetic susceptibility to alcohol-related complications after MBS [194]. Many institutions use structured risk stratification to inform patient selection and surgical preparation, classifying individuals as low, moderate, or high risk. For those in the moderate- and high-risk categories, additional protective measures are advised, including behavioral contracts that formalize commitments to abstinence, requirements for a minimum duration of documented sobriety, often at least one year, when relapse risk decreases, and enhanced informed consent that explicitly addresses altered alcohol pharmacokinetics and the potential for neurological and hepatic injury [195]. While active SUD is generally considered a contraindication to surgery, patients in stable recovery may achieve favorable outcomes when supported through coordinated, multidisciplinary care [194]. Significantly, this risk stratification must also dictate a more intensive nutritional surveillance pathway; for high-risk drinkers, it is recommended to monitor laboratory levels for B1 (thiamine), B12, folate, ferritin, and Vitamin D every 3 months during the first postoperative year (at months 3, 6, 9, and 12), transitioning to every 6 months in the second year, and annually thereafter to prevent the onset of irreversible metabolic or neurological complications [154,196,197].

### 6.2. Postoperative Screening Tools

Routine postoperative screening for substance use is essential, particularly during the first three years following surgery, when the risk of developing new-onset AUD is highest [32]. Because stigma and underreporting can limit the accuracy of self-disclosure, the use of standardized, validated screening instruments is strongly recommended. Commonly used tools include the Alcohol Use Disorders Identification Test (AUDIT), a 10-item measure of alcohol consumption and related harms, and its abbreviated version, the AUDIT-C, which focuses on consumption and is well suited for brief clinical encounters [198]. Drug misuse can be assessed using the 10-item Drug Abuse Screening Test (DAST-10), with scores of three or higher indicating the need for further evaluation, while the TAPS tool enables rapid screening across tobacco, alcohol, prescription medications, and illicit substances within a single interaction [198,199]. More recently, digital platforms such as ATTAIN have demonstrated superior detection rates compared with routine office-based care, likely because patients feel more comfortable disclosing sensitive behaviors in web-based formats [200].

### 6.3. Nutritional Interventions and Supplementation

A key nutritional focus in the postoperative management of bariatric patients is the prevention of thiamine deficiency and its potentially severe neurological consequences, particularly in the context of the combined effects of alcohol use and malabsorption [18]. Care is individualized according to clinical risk. All patients should receive routine multivitamin supplementation providing at least 1.2–1.5 mg of thiamine daily, with additional oral thiamine (100–300 mg per day) recommended for those who continue to consume alcohol [135]. In situations of acute risk, such as persistent vomiting or severe malnutrition, parenteral thiamine at a dose of 300 mg daily should be administered prior to any carbohydrate intake [137]. When Wernicke’s encephalopathy is suspected, immediate treatment with high-dose intravenous thiamine (500 mg three times daily) is required, followed by ongoing oral replacement. In addition to micronutrient management, maintaining adequate protein intake (1.2–1.5 g/kg/day) is essential to prevent sarcopenia [136,137,201]. Patients are also counseled to separate fluid and solid intake, avoid carbonated beverages, and limit high-glycemic foods to minimize dumping syndrome and reduce the risk of maladaptive eating behaviors [202,203]. Although these recommendations are supported by clinical guidelines and observational evidence, randomized controlled trials specifically evaluating nutritional and behavioral interventions for alcohol and substance use in post-bariatric populations remain limited.

### 6.4. Behavioral, Multidisciplinary, and Digital Support Strategies

Behavioral care must extend beyond simple abstinence messages. Motivational interviewing and cognitive behavioral therapy help patients recognize their heightened biological sensitivity to alcohol after surgery [204]. Bariatric-specific support groups facilitate discussion of addiction transfer and provide strategies for navigating alcohol-related social situations [205]. Behavioral contracts initiated preoperatively should be revisited postoperatively to reinforce long-term commitments [194].

Optimal outcomes are best achieved through coordinated, team-based care, as advocated by the ASMBS Integrated Health Section. This multidisciplinary model typically includes bariatric surgeons, registered dietitians, behavioral health professionals, and addiction specialists capable of managing complex alcohol or opioid use disorders, including the use of medication-assisted treatment when indicated [206,207,208,209]. Integrated clinical programs that deliver these services within a single institution or via shared telehealth networks have demonstrated improved continuity of care and more comprehensive management of substance-related complications [210].

Telehealth and digital health technologies further enhance long-term surveillance and patient engagement, particularly during the high-risk postoperative window of two to five years [211]. Web-based platforms, mobile applications, and tailored text messaging interventions provide anonymity, standardized education, and real-time behavioral support, which may increase patient disclosure and adherence to treatment recommendations [212]. Together, behavioral counseling, multidisciplinary care, and digital solutions form a cohesive framework for mitigating substance-related risks and supporting sustained postoperative success. To date, most intervention strategies are derived from multidisciplinary clinical practice and studies in general addiction populations, as randomized controlled trials specifically targeting substance use disorders in post-bariatric cohorts are scarce.

## 7. Conclusions

The long-term success of bariatric surgery extends far beyond the operating room, requiring proactive, multidisciplinary management of metabolic, nutritional, and behavioral risks. Postoperative alcohol and substance use, amplified by altered pharmacokinetics and neurobiological reward shifts, can undermine weight loss, increase liver injury, and contribute to psychological distress. These risks are often delayed, emerging years after the “honeymoon” phase of rapid weight loss, and can be exacerbated by maladaptive eating behaviors or “addiction transfer,” where substances replace food as a primary reward.

Despite the recognized independent effects of surgery or substance use on nutrition, there remains a paucity of research examining how alcohol and drug disorders interact with post-bariatric malabsorption to deplete critical micronutrients such as thiamine, vitamin B12, and iron. Addressing this gap through integrated, longitudinal screening and targeted interventions is essential to prevent irreversible complications and optimize patient outcomes. By viewing AUD and SUD as chronic relapsing brain diseases with neurobiological, metabolic, behavioral, and psychosocial dimensions, rather than lifestyle choices, clinicians can better safeguard the transformative benefits of bariatric medicine and reduce stigma in postoperative care.

## Figures and Tables

**Table 1:** Substance use after BS: prevalence, mechanisms and clinical implications.

Substance	Prevalence in Bariatric Population	Key Mechanisms Post-MBS	Impact on Weight & Metabolic Outcomes
**Alcohol**	Delayed onset with progressive increase; ~7–10% by 2 years, rising to ~20% by 5–7 years post-RYGB [32,35,122]	Altered pharmacokinetics, addiction transfer, neurohormonal changes [35,87,89]	WR, hypoglycemia, hepatic injury [26,28,31,32]
**Opioids**	High pre-op use; ↑ 13–18% post-op [99,100]	Persistent pain syndromes, altered reward pathways [100]	Impaired weight loss, reduced physical activity [98,102,103]
**Cannabis**	~6–8% pre-op; increasing post-op [97]	Appetite stimulation, altered pain control [104]	Potential caloric excess, reduced adherence [97,105]

Abbreviations: MBS, metabolic and bariatric surgery; RYGB, Roux-en-Y gastric bypass; WR, weight regain; ↑, increase.

**Table 2:** Management of Thiamine Deficiency in Post-Bariatric Patients, Including Alcohol-Related Risk.

Severity of Deficiency	Clinical Entity [134,135]	Key Clinical Features [128,129]	Typical Triggers in Post-Bariatric Patients [135,136]	Management [137]
**Mild/Subclinical**	Early thiamine depletion	Fatigue, anorexia, irritability, peripheral neuropathy, vague cognitive changes	Poor oral intake, vomiting, supplement nonadherence, early alcohol use	Oral: 300 mg/day for 3–5 days, then 100 mg/day maintenance. Multivitamin supplementation; dietary optimization
**Moderate/Acute**	Wernicke’s encephalopathy	Confusion, ataxia, ophthalmoplegia/nystagmus (often incomplete triad)	Heavy or binge alcohol use, prolonged vomiting, rapid weight loss, dehydration	Parenteral (IV/IM): 300 mg daily; must be given before any carbohydrate (e.g., IV glucose). Consider magnesium replacement and correction of other micronutrient deficiencies
**Severe/Chronic**	Korsakoff syndrome	Profound memory impairment, confabulation, executive dysfunction; often irreversible	Long-standing deficiency, delayed treatment, continued alcohol use	High-Dose IV: 500 mg three times daily initially, followed by long-term oral replacement; neurorehabilitation and strict alcohol abstinence

Abbreviations: IV, intravenous; IM, intramuscular.

## Data Availability

No new data were created or analyzed in this study. Data sharing is not applicable to this article.
